# E2F7 overexpression leads to tamoxifen resistance in breast cancer cells by competing with E2F1 at miR-15a/16 promoter

**DOI:** 10.18632/oncotarget.5128

**Published:** 2015-09-12

**Authors:** Junjun Chu, Yinghua Zhu, Yujie Liu, Lijuan Sun, Xiaobin Lv, Yanqin Wu, Pengnan Hu, Fengxi Su, Chang Gong, Erwei Song, Bodu Liu, Qiang Liu

**Affiliations:** ^1^ Breast Tumor Center, Sun Yat-sen Memorial Hospital, Sun Yat-sen University, Guangzhou 510120, China; ^2^ Key Laboratory of Malignant Tumor Gene Regulation and Target Therapy of Guangdong Higher Education Institutes, Sun Yat-sen Memorial Hospital, Sun Yat-sen University, Guangzhou 510120, China; ^3^ Key Laboratory of Gene Engineering of Ministry of Education, State Key Laboratory of Biocontrol, School of Life Sciences, Sun Yat-sen University, Guangzhou 510275, China

**Keywords:** breast cancer, tamoxifen resistance, E2F7, miR-15a/16, prognostic marker

## Abstract

About 50–70% of breast cancers are estrogen receptor α (ERα) positive and most of them are sensitive to endocrine therapy including tamoxifen. However, one third of these patients will eventually develop resistance and relapse. We found that the expression of miR-15a and miR-16 were significantly decreased in tamoxifen resistant ER positive breast cancer cell lines. Exogenous expression of miR-15a/16 mimics re-sensitized resistant cells to tamoxifen by inhibiting Cyclin E1 and B cell lymphoma-2 (Bcl-2) to induce cell growth arrest and apoptosis respectively. Further, we identified that a repressive member of E2F family, E2F7, was responsible for the suppression of miR-15a/16 cluster by competing with E2F1 for E2F binding site at the promoter of their host gene DLEU2. Moreover, high expression of E2F7 is correlated with high risk of relapse and poor prognosis in breast cancer patients receiving tamoxifen treatment. Together, our results suggest that overexpression of E2F7 represses miR-15a/16 and then increases Cyclin E1 and Bcl-2 that result in tamoxifen resistance. E2F7 may be a valuable prognostic marker and a therapeutic target of tamoxifen resistance in breast cancer.

## INTRODUCTION

Oestrogen signaling plays a central role in female physiology through its effects on critical cellular processes, including cell proliferation and survival. ERα is the main receptor of oestrogen in breast tissues. About 50-70% of breast cancer patients are classified as ERα positive and interference with ERα signaling has been an effective treatment strategy for over a century [1]. Tamoxifen has been the most widely used endocrine therapy in ERα positive breast cancer patients for more than 30 years [2]. However, one-third of ERα tumors will eventually develop resistance and relapse, presenting a huge challenge for the cure of breast cancer.

Various mechanisms have been proposed to explain tamoxifen resistance, including loss of ERα expression [3] and cross-talk between ER and receptor tyrosine kinase signaling [4, 5]. Because of the complexity of tamoxifen resistance, the underlying mechanisms are not fully understood yet. Recent studies reported that abnormal expression of miRNAs plays a role in tamoxifen resistance. For instance, it was shown that up-regulated miR-221/222 induced tamoxifen resistance by targeting p27 [6]; miR-451 was responsible for resistance to tamoxifen by targeting 14-3-3 zeta [7]; re-expression of miR-375 reversed both tamoxifen resistance and EMT phenotypes [8]. MiR-15a/16 cluster, located at chromosomal region 13q14 and frequently deleted in cancer, are important miRNAs that act as tumor suppressors in chronic lymphocytic leukemia (CLL) and other malignancies [9, 10]. It was reported that exogenous expression of HER2Δ16, a mutant form of HER2 in MCF7 cells, suppressed miR-15a/16 and induced endocrine resistance [11]. However, the mechanism is unclear and whether miR-15a/16 have a role in the tamoxifen resistance of HER2-negative ERα-positive cancers remains unknown.

The E2F family of transcription factors (E2F 1-8) is known to regulate genes involved in cell proliferation, differentiation and apoptosis. E2F1-3 are known to be transcription activators, while other family members, including E2F7, are known as transcription repressors. Chromatin Immunoprecipitation sequencing (CHIP-seq) analysis revealed that E2F7 binds preferentially to the genomic sites closely resemble the E2F consensus site [12]. Some reports have linked abnormal expression of E2F7 with cancer, for instance, increased E2F7 expression in cutaneous SCC [13] and its decreased expression in ovarian cancer [14]. However, the link between abnormal expression of E2F7 and breast cancer remain unclear.

In the current study, we found that miR-15a/16 were down-regulated in tamoxifen-resistant breast cancer cells. Exogenous expression of miR-15a/16 in tamoxifen resistant cells induced cell cycle arrest and apoptosis by inhibiting Cyclin E1 and Bcl-2. Further, we identified that elevated E2F7 was responsible for transcriptional repression of miR-15a/16 cluster in tamoxifen-resistant breast cancer cells. Silencing E2F7 re-sensitizes breast cancer cells to tamoxifen through up-regulation of miR-15a/16. Finally, using several publicly available datasets, we found that high E2F7 expression is associated with higher relapse rate and poor prognosis of breast cancer patients receiving tamoxifen treatment.

## RESULTS

### ERα positive breast cancer cells with long-term exposure to tamoxifen acquired resistance to tamoxifen- induced proliferation inhibition and apoptosis

**Figure 1:**
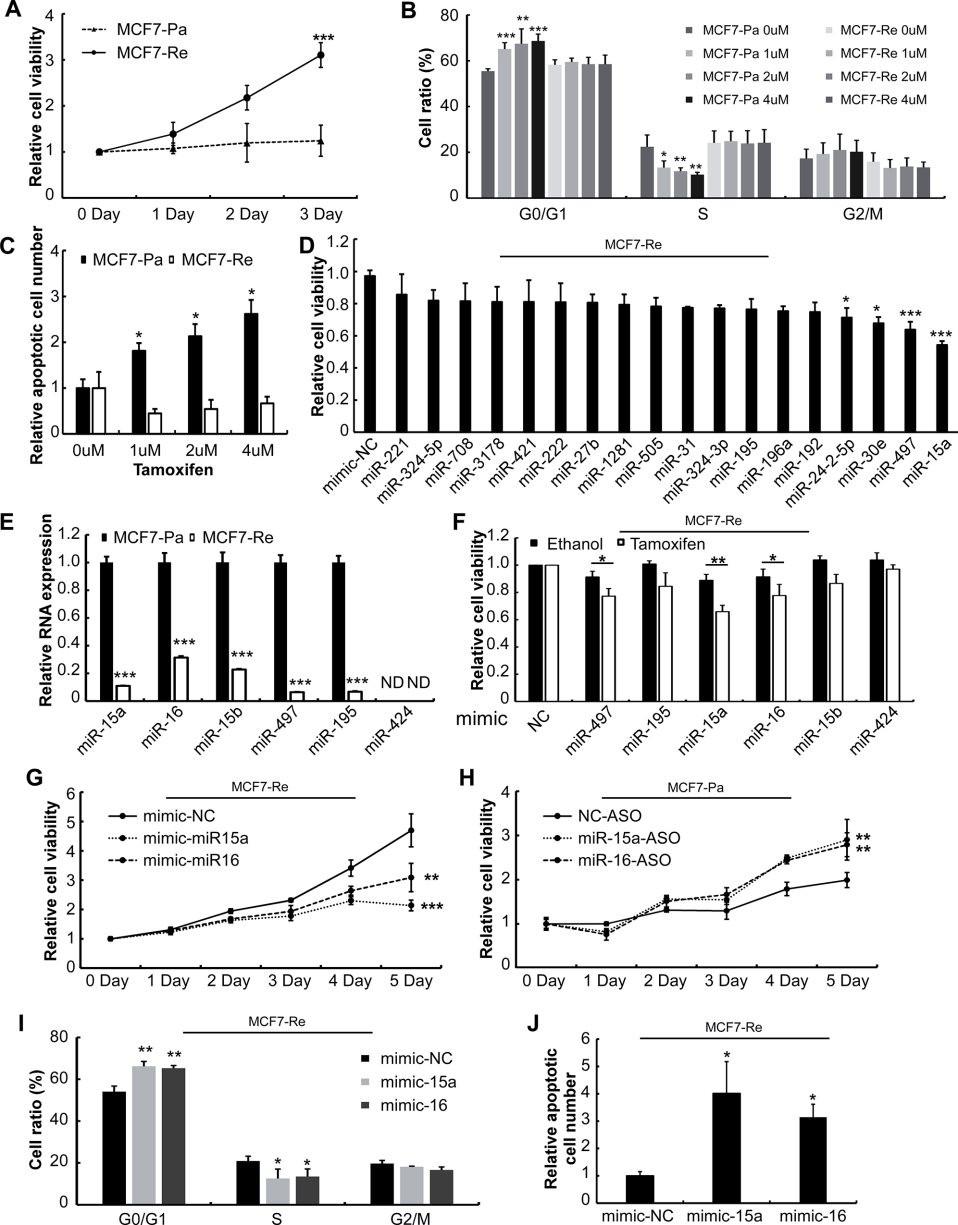
Screening for functional miRNAs in tamoxifen resistance **A.** Proliferation of MCF7-Pa and MCF7-Re were determined by MTT under 1 uM Tamoxifen treatment. **B.** After 3 days' treatment with 0-4 uM tamoxifen, cell cycle was analyzed by flow cytometry. The bar chart represents the percentage of cells in G1/G0, S, or G2/M phase. **C.** Apoptotic cells number was measured by flow cytometry. **D.** MCF7-Re cells viability were measured by MTT after transfection of miRNA mimics under 1 uM tamoxifen treatment. **E.** Expression of miR-15a family miRNAs in MCF7-Pa and MCF7-Re cells were detected by qPCR (ND: Not Detected). **F.** MCF7-Re cells viability were measured by MTT after transfected miRNA mimics under ethanol or 1 uM tamoxifen treatment. **G.** MCF7-Re cells proliferation were determined by MTT after transfected with miRNA mimics under 1 uM tamoxifen. Cell cycle **I.** and apoptosis **J.** were measured after 3days transfection and treatment with 1 uM tamoxifen. **H.** MCF7-Pa cells proliferation were determined by MTT after transfected with miRNA ASOs under 1 uM tamoxifen. (*p < 0.05, **p < 0.01, ***p < 0.001.)

In order to develop an *in vitro* model of tamoxifen resistance, we developed a tamoxifen resistant cell line model similar to previous studies [15,16]. ERα positive and tamoxifen sensitive breast cancer cell lines MCF7 and T47D were cultured in phenol-free media supplied with charcoal-stripped bovine serum (cFBS) and exposed to increased concentration of tamoxifen up to 1 μM for 1 year. Tamoxifen inhibits MCF7 cell proliferation by inducing G1/G0 arrest of cell cycle and causes cell death [17, 18]. But after one year exposure of tamoxifen, MCF7 parental (MCF7-Pa) cells and T47D parental (T47D-Pa) cells acquired resistance to tamoxifen, and became MCF7-Resistant (MCF7-Re) and T47D resistant (T47D- Re) cells. To verify tamoxifen resistance of MCF7-Re and T47D-Re cells, we performed MTT assay to measure cell proliferation. The viability of MCF7-Re and T47D-Re cells in the presence of 1 μM tamoxifen was significantly higher than that of their Parental cells (Figure 1A, Figure S2A). Further, the induced cell cycle arrest and apoptosis of MCF7-Re cells under 1-4 μM tamoxifen were also significantly lower than that of MCF7-Pa cells (Figures 1B, 1C). These data demonstrated that the MCF7-Re and T47D-Re cell lines, cultured by long time exposure to tamoxifen, acquired resistance to tamoxifen.

### Suppressed expression of miR-15a/16 causes tamoxifen resistance of MCF7-Re and T47D-Re cells

Affymetrix GeneChip® miRNA 3.0 microarray was used to examine the miRNAs differentially expressed between MCF7-Pa and MCF7-Re cells. With a cut-off value of 2 fold increase or decrease, 18 miRNAs were down-regulated and 15 were up-regulated (Table 1). Down-regulated mature miRNAs were validated by quantitative real-time PCR (qPCR) (Figure S1). To identify the miRNAs that are responsible for tamoxifen resistance, miRNA mimics of the 18 down-regulated miRNAs were used for functional screening. The results indicated that transfection of miR-15a (*p* < 0.001) and miR-497 (*p* < 0.05) mimics re-sensitized MCF7-Re cells to tamoxifen treatment (Figure 1D). Interestingly, miR-15a and miR-497 belong to the miR-15a miRNA family and have similar sequences. We further found that most of the miR-15a family members, including miR-497, miR-195, miR-15a, miR-16, and miR-15b, were significantly down-regulated in MCF7-Re cells (Figure 1E). Exogenous expression of those miRNAs could re-sensitize MCF-Re cells to tamoxifen at different extent (Figure 1F).

**Table 1:** List of differentially expressed microRNAs in MCF7-Re compared with MCF7-pa cells

miRNA	Fold change (Re/Pa)
**Top down regulated (FC < 0.5)**	
hsa-mir-497	0.15
hsa-mir-1281	0.16
hsa-mir-222	0.24
hsa-mir-221	0.25
hsa-mir-195	0.26
hsa-mir-27b	0.27
hsa-mir-24-2-5p	0.34
hsa-mir-324-3p	0.38
hsa-mir-30e	0.41
hsa-mir-505	0.41
hsa-mir-708	0.41
hsa-miR-196a	0.42
hsa-mir-31	0.42
hsa-miR-3178	0.43
hsa-mir-421	0.43
hsa-mir-192	0.47
hsa-mir-15a	0.47
hsa-mir-324-5p	0.49
**Top up regulated (FC > 2)**	
hsa-mir-3201	4.89
hsa-mir-4521	3.34
hsa-mir-720	2.59
hsa-mir-4492	2.54
hsa-mir-455	2.43
hsa-mir-375	2.29
hsa-mir-149	2.19
hsa-mir-378c	2.19
hsa-mir-4728	2.14
hsa-mir-3200	2.14
hsa-mir-503	2.13
hsa-mir-4508	2.10
hsa-mir-4507	2.09
hsa-mir-4449	2.09
hsa-mir-3135b	2.07

Abbreviation: has: Homo sapiens; miR: microRNA; FC: Fold change Re: Resistant; Pa: parent

Among miR-15a family miRNAs, miR-15a/16 were highly efficient to restore tamoxifen sensitivity and were transcribed from the same cluster. In another tamoxifen resistant ER positive cell line (T47D-Re), miR-15a/16 were also significantly down-regulated compared with T47D-Pa cells (Figure S2B). Transfection of miR-15a/16 mimics reduced viability of MCF7-Re and T47D-Re cells (Figure 1G, Figure S2C) and induced cell cycle arrest (Figure 1I, Figure S2D) and apoptosis (Figure 1J, Figure S2E) under tamoxifen treatment. On the other hand, silencing miR-15a/16 expression by antisense oligos (ASOs) reduced tamoxifen sensitivity of MCF7-Pa cells (Figure 1H). Together, these data indicated that miR-15a/16 were suppressed in tamoxifen resistant breast cancer cells, and exogenous expression of miR-15a/16 mimics re-sensitized resistant cells to tamoxifen.

### MiR-15a/16 reduce tamoxifen resistance by inhibiting Bcl-2 and Cyclin E1

Previous studies showed that Bcl-2 [9, 11] and Cyclin E1 [19] were target genes of miR-15a/16. Bcl-2 is an important player in multidrug resistance in different types of cancer and miR-15a/16 were shown to induce apoptosis by targeting Bcl-2 in CLL [20]. It was also reported that high expression of Cyclin E1 was a strong predictor of endocrine therapy failure in human breast cancer [21].

**Figure 2:**
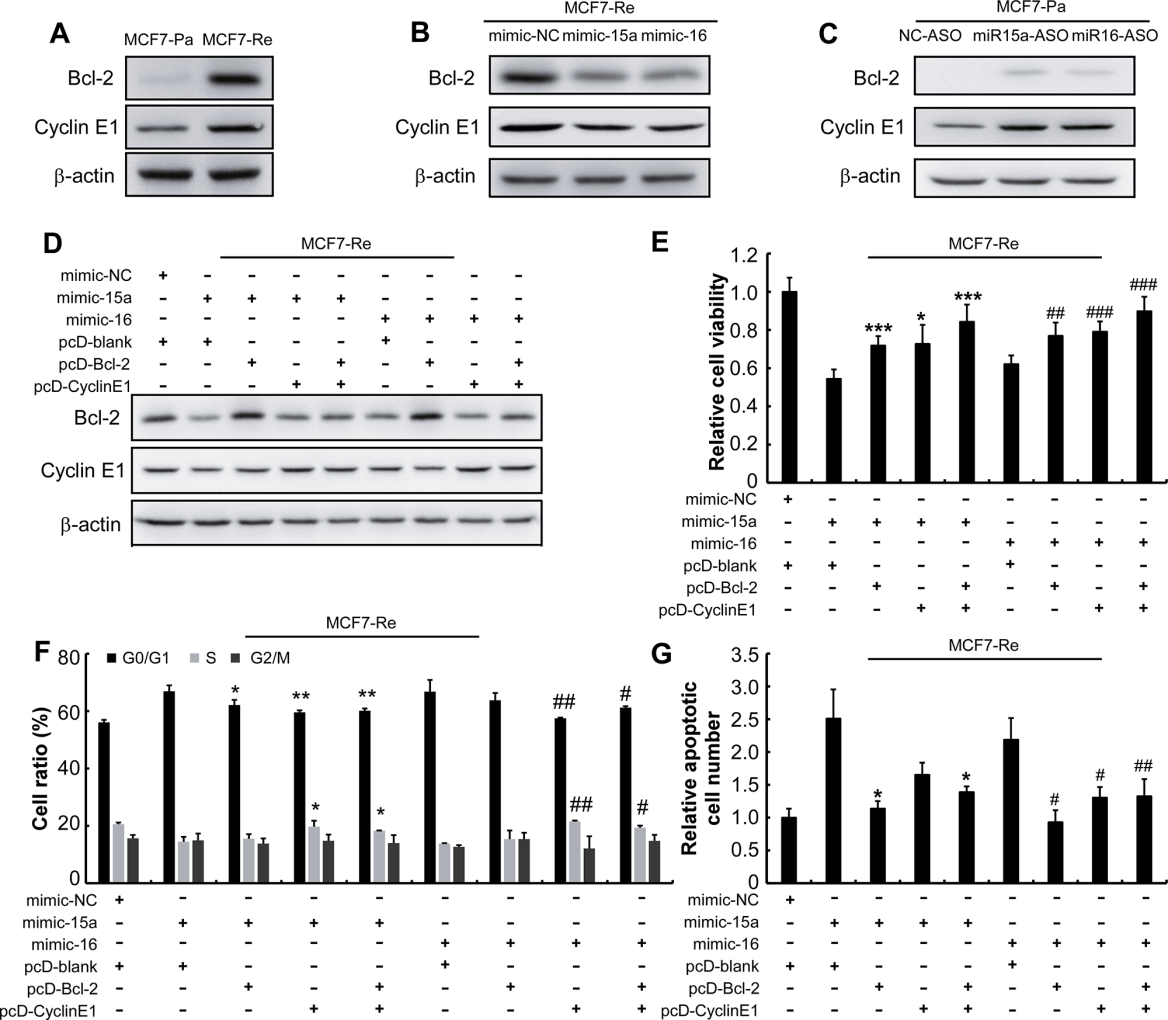
MiR-15a/16 reduce the resistance of MCF7-Re cells to tamoxifen by inhibiting Bcl-2 and Cyclin E1 **A.** Western blot analysis for Bcl-2 and Cyclin E1 in MCF7-Pa and MCF7-Re cells. β-actin was used as an internal control, hereafter. **B.** Western blot of Bcl-2 and Cyclin E1 in the MCF7-Re cells that were transfected with miRNA mimics and **C.** in the MCF7-Pa cells that transfected with miRNA ASOs. **D.** Western blot was performed to determine the expression of Bcl-2 and Cyclin E1 in the MCF7-Re cells that were transfected with miRNA mimics alone or co-transfected with pcDNA6B plasmid vector or pcDNA6B cloned with a CDS of Bcl-2 and Cyclin E1. And **E.** cell viability was determined by MTT, **F.** cell cycle, **G.** apoptosis were determined by flow cytometry. *p < 0.05, **p < 0.01, ***p < 0.001, versus cells transfected with miR-15a mimic and vector; #*p* < 0.05, ##*p* < 0.01, ###*p* < 0.001, versus cells transfected with miR-16 mimic and vector.

In light of the previous findings, we examined whether Bcl-2 and Cyclin E1 were the functional targets of miR-15a/16 in regulating tamoxifen sensitivity. We found that both Bcl-2 and Cyclin E1 protein expression were up-regulated in MCF7-Re and T47D-Re cells (Figure 2A, Figure S3A). Transfection with mimics of miR-15a and miR-16 reduced Bcl-2 and cyclin E1 protein expression in MCF7-Re cells (Figure 2B), while miR-15a and miR-16 ASOs increased Bcl-2 and Cyclin E1 expression in MCF7-Pa cells (Figure 2C).

To further evaluate whether miR-15a/16-inhibited expression of Cyclin E1 and Bcl-2 is responsible for tamoxifen resistance, we co-transfected MCF7-Re cells with mimics of miR-15a or miR-16 and a pcDNA6B vector carrying Bcl-2 coding sequence (CDS) or Cyclin E1 CDS. Western blotting demonstrated that co-transfection of pcDNA6B vectors carrying Bcl-2 or Cyclin E1 expression cassette restored the protein expression of Bcl-2 or Cyclin E1 respectively. (Figure 2D). Moreover, restoring Bcl-2 or Cyclin E1 expression in MCF7-Re cells transfected with miR15a/16 mimics recapitulated tamoxifan-resistant phenotype of MCF7-Re cells, including enhanced proliferation (Figure 2E), reduced cell cycle arrest (Figure 2F) and reduced apoptosis (Figure 2G). Moreover, re-expression of both Bcl-2 and Cyclin E1 almost fully restored tamoxifen-resistance (Figure 2E–2G) when miR-15a/16 mimics were co-transfected, suggesting Bcl-2 and Cyclin E1 work synergistically to confer tamoxifen resistance in breast cancer cells, but not being redundant to each other. Together, these results demonstrate that miR-15a/16 re-sensitize tamoxifen resistant cells to tamoxifen by targeting Bcl-2 and Cyclin E1.

### MiR-15a/16 cluster is transcriptionally inhibited in tamoxifen resistant cells

**Figure 3:**
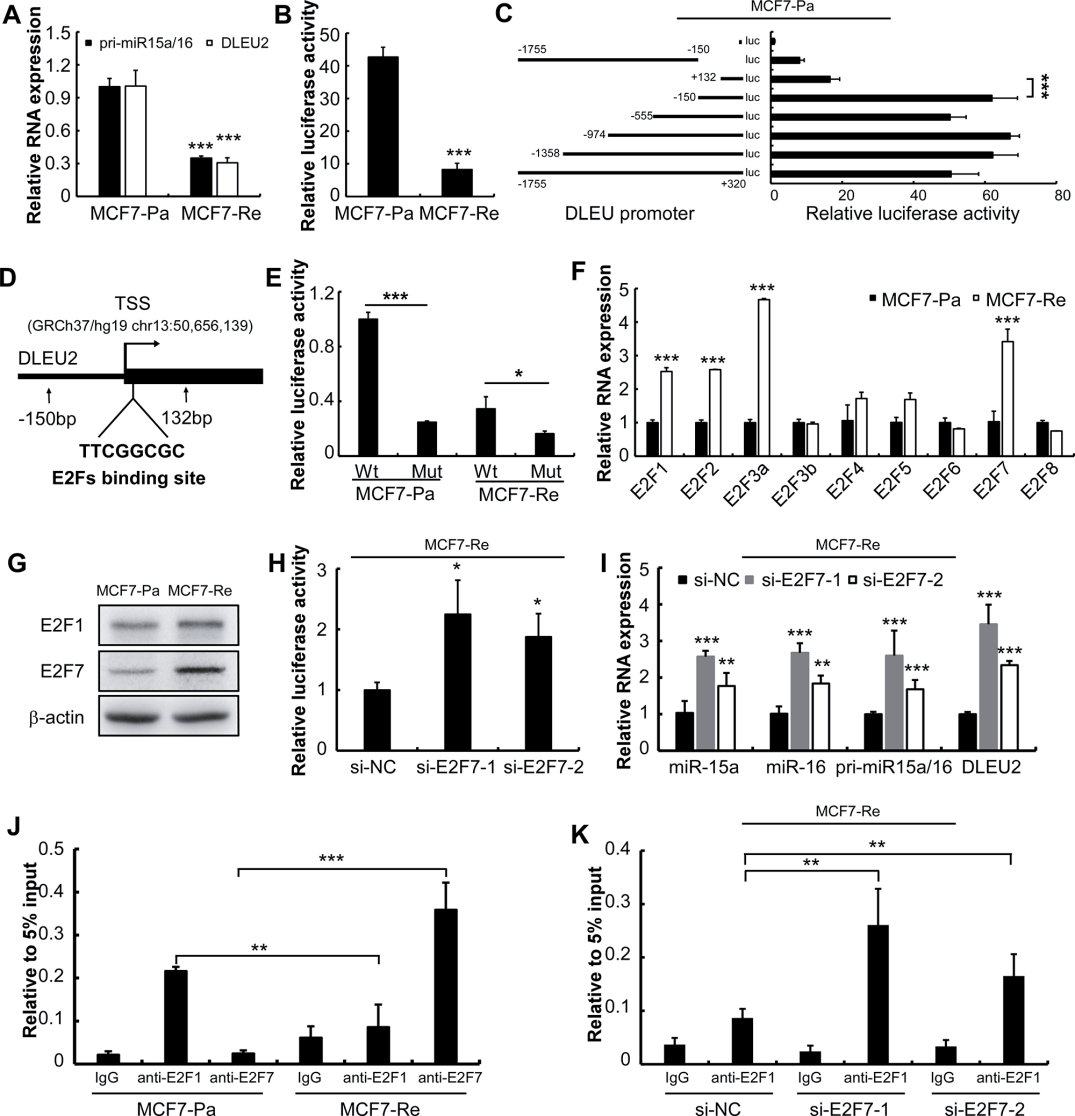
E2F7 suppresses miR-15a/16 expression by competing E2F1 binding site in MCF7-Re cells **A.** qPCR of pri- miR-15a/16 and the host gene DLEU2 in MCF7-Re cells compared with MCF7-Pa cells. **B.** Luciferase reporter assay for MCF7-Pa and MCF7-Re cells transfected with reporter plasmids containing DLEU2 promoter. **C.** Luciferase reporter assay for MCF7-Pa cells transfected with reporter plasmids containing truncated DLEU2 promoters. **D.** Diagram of DLEU2 promoter and predicted E2F binding site. **E.** luciferase reporter assay for wild type and E2F binding site mutant DLEU2 promoters' (−320bp ~ 150bp) activity in MCF7-Pa and MCF7-Re cells. **F.** qPCR of all E2F family members in MCF7-Re cells compared with MCF7-Pa cells. **G.** Western blot of E2F1 and E2F7 protein in MCF7-Pa and MCF7-Re cells. **H.** Luciferase reporter assay for MCF7-Re cells transfected with full length DLEU2 promoter after 24 hours transfection of E2F7 siRNAs. **I.** qPCR of mature miR-15a, miR-16, pri-miR-15a/16 and DLEU2 in MCF7-Re cells after transfected with E2F7 siRNAs. **J.** CHIP was performed to measure the binding activity of E2F1 and E2F7 to DLEU2 promoter. Primer was designed to detect predicted E2F binding site. Data are normalized to 5% input for each cell type. **K.** CHIP was performed to measure the binding activity of E2F1 to DLEU2 promoter after transfection of E2F7 siRNAs in MCF7-Re cells. (*p < 0.05, **p < 0.01, ***p < 0.001.)

The miR-15a and miR-16 cluster resides at chromosome 13q14.3, a genomic region that is frequently deleted in CLL and a subset of mantle cell lymphoma [9, 10]. Nevertheless, deletion of this region is rare in breast cancer patients [11]. MiR-15a and miR-16 are encoded within an intronic region of the non-coding DLEU2 gene in human and share the same promoter of DLEU2. To explore how miR-15a/16 are down-regulated in tamoxifen resistant cells, we measured the expression of the primary transcript (pri-miR-15a/16) and DLEU2 in MCF7-Pa and MCF7-Re cells. Both of them were significantly down-regulated at a similar ratio in MCF7-Re (Figure 3A). Pri-miR-15a/16 and DLEU2 were also down-regulated in T47D-Re cells compared with T47D-Pa cells (Figure S2B). Furthermore, the transcriptional activity of DLEU2 promoter is significantly down-regulated in MCF7-Re cells detected by luciferase reporter assay (Figure 3B). These results indicated that miR-15a/16 cluster is transcriptionally inhibited in MCF7-Re cells.

### E2F7 suppresses miR-15a/16 expression by competing with E2F1

Transcription factors are the most important regulators of transcription. Therefore, we investigated which transcription factor is responsible for the down-regulation of miR-15a/16 cluster. To narrow down the targets, we constructed a series of sequentially deleted DLEU2 promoters from −1755 ~ +320 to +132 ~ +320, relative to transcription start site (TSS) (GRCh37/hg13:50,656,139). The transcriptional activity of +132 ~ +320 constructs was significantly decreased compared with that of −150 ~ +320 (Figure 3C), whereas other truncated promoter constructs showed similar transcriptional activities. This result suggested the presence of key regulatory elements between −150bp to +132 bp of the promoter. Indeed, a consensus E2F binding sequence was found in this region using TFSEARCH (http://www.cbrc.jp/research/db/TFSEARCH.html) [22] (Figure 3D). Furthermore, mutation of this E2F binding site significantly decreased the transcription activity in MCF7-Pa (*p* < 0.001) and MCF7-Re (*p* < 0.05) cells to different extents (Figure 3E). This is in agreement with the findings that the promoter is more actively transcribed in MCF7-Pa cells than in MCF-Re cells.

E2F1 has been reported to stimulate miR-15a/16 cluster expression in osteosarcoma cell line U2OS and lung adenocarcinoma cell line H1299 by binding to the same site of DLEU2 promoter [19]. Similar to these findings, we showed that silencing E2F1 indeed decreased the expression of mature miR-15a/16, pri-miR-15a/16 and DLEU2 in MCF7-Pa cells (Figure S4 A.B). However, we found that the protein level of E2F1 was similar between MCF7-Re cells and MCF7-Pa cells (Figure 3F/3G), suggesting the existence of another regulator.

It is known that E2F family members share very similar DNA binding sequence, but their functions are widely different and the regulation between E2F genes is very complex [23]. Previous study demonstrated that E2F6 can repress gene transcription by interacting with E2F1 [34]. Therefore, we examined whether other E2F family members participate in the transcriptional regulation of miR-15a/16 cluster. Several activators (E2F1, E2F2, E2F3a) were significantly up-regulated in MCF7-Re cells than MCF7-Pa cells, while the only significantly differentially expressed repressor was E2F7 (Figure 3F). Western blot confirmed that E2F7 protein expression is significantly up-regulated in MCF7-Re and T47D-Re cells than their parent cells (Figure 3G, Figure S3B). Knocking down of E2F7 by siRNAs restored the wild type DLEU2 promoter activity (Figure 3H) and up-regulated the expression of mature miR-15a/16, pri-miR-15a/16 and DLEU2 (Figure 3I, Figure S3C).

Choromatin immunoprecipitaion (ChIP) of DLEU2 promoter followed by qPCR was performed to examine whether E2F1 and E2F7 bound to DLEU2 promoter. Interestingly, the amount of E2F1 at E2F binding site in DLEU2 promoter was significantly reduced in MCF7-Re and T47D-Re cells, compared to their parent cells, while E2F7 presence was significantly increased at the same E2F binding site (Figure 3J, Figure S3D). This suggested that in tamoxifen resistant cells, up-regulated E2F7 effectively competed with E2F1 for the E2F binding site located at DLEU2 promoter, and transcriptionally silenced DLEU2-miR-15b/16 expression. To further validate this hypothesis, we knocked down E2F7 in MCF7-Re and T47D-Re cells by siRNAs. Indeed, silencing E2F7 in MCF7-Re and T47D-Re cells significantly enhanced E2F1 binding at DLEU2 promoter (Figure 3K, Figure S3E).

In conclusion, E2F7 is up-regulated in tamoxifen resistant cells and repressed miR-15a/16 cluster expression by competing with E2F1 for the E2F binding site at DLEU2 promoter.

### Silencing of E2F7 in tamoxifen resistant cells re-sensitizes cells to tamoxifen

**Figure 4:**
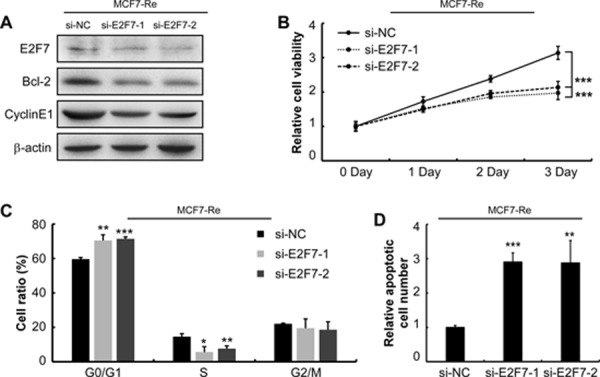
Silencing of E2F7 in MCF7-Re cells sensitizes cells to tamoxifen **A.** E2F7, Bcl-2 and Cyclin E1 protein expression was determined by western blotting in MCF7-Re cells that were transfected with E2F7 siRNAs. **B.** proliferation rate of MCF7-Re cells under 1 uM tamoxifen were measured every day after transfected with E2F7 siRNAs by MTT assay. **C.** MCF7-Re cells were transfected with E2F7 siRNAs and treated with 1 uM tamoxifen for 3days and cell cycle distribution was monitored by flow cytometry, **D.** apoptosis was detected by flow cytometry. (*p < 0.05, **p < 0.01, ***p < 0.001.)

To investigate the effect of high E2F7 expression in tamoxifen resistant cells, we silenced E2F7 by siRNAs in MCF7-Re and T47D-Re cells. Bcl-2 and Cyclin E1 protein expression were decreased after silencing of E2F7 (Figure 4A, Figure S5A). In agreement, silencing E2F7 re-sensitized MCF7-Re to tamoxifen, shown by cell viability (Figure 4B, Figure S5B), cell cycle arrest (Figure 4C, Figure S5C) and cell apoptosis (Figure 4D, Figure S5D). These data indicated that highly expressed E2F7 is key to tamoxifen resistance of cells.

### Higher expression of E2F7 is significantly correlated with poor prognosis and higher risk of relapse in tamoxifen treated patients

Previous study identified that E2F7 was overexpressed in cutaneous squamous cell carcinomas (SCC), and could suppress proliferation and apoptotic responses. Inhibition of E2F7 in a SCC cell line sensitized the cells to UV-induced apoptosis and doxorubicin induced apoptosis [13]. However, another study indicated that down-regulation of E2F7 may contribute to mechanisms underlying platinum resistance in 77 ovarian cancer patients [14]. The expression and function of E2F7 in breast cancer patients especially the relationship with tamoxifen resistance has never been reported.

**Figure 5:**
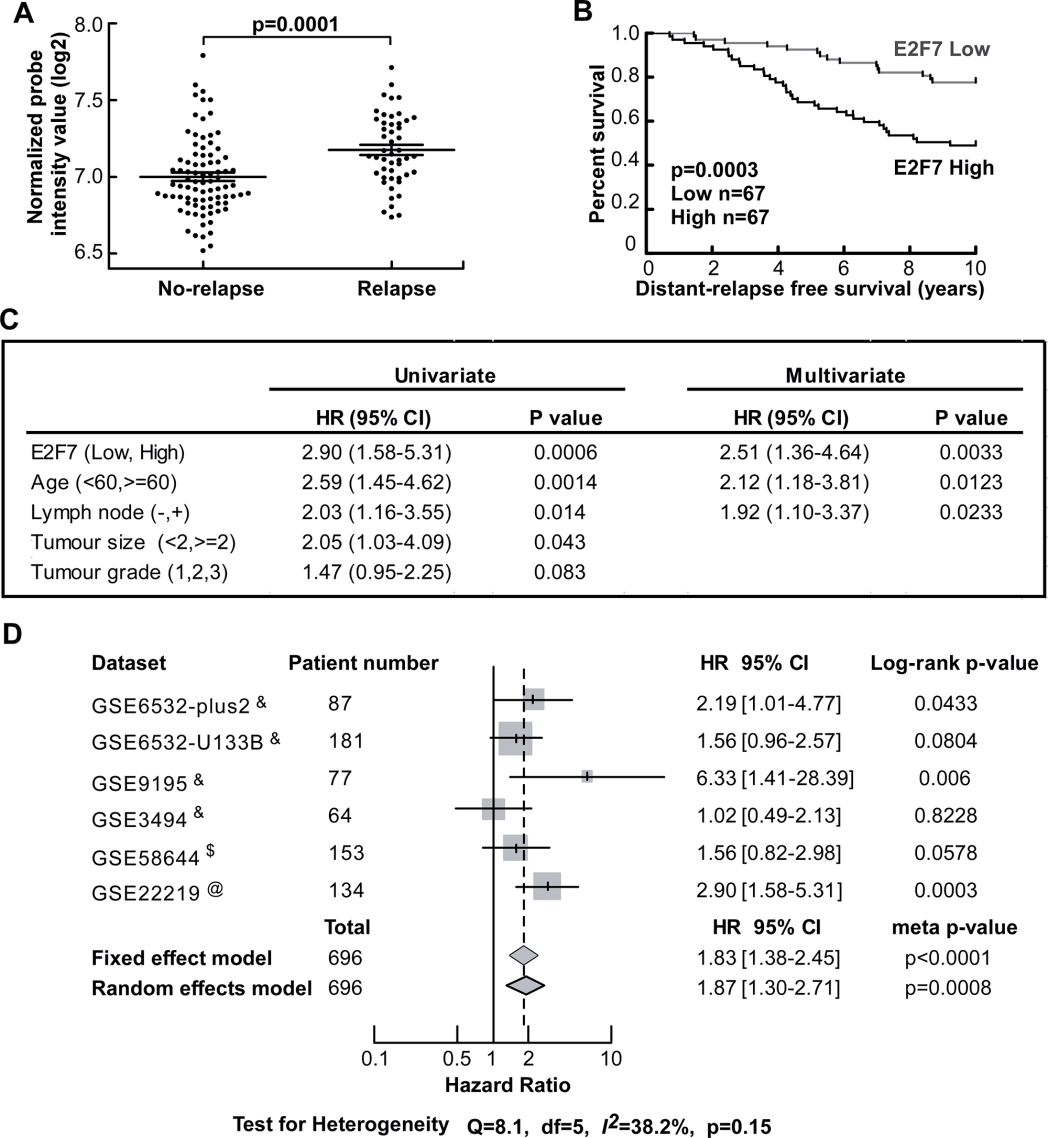
Higher expression of E2F7 is significantly correlated with poorer prognosis and higher risk of relapse in tamoxifen treated patients **A.** Analysis of 134 ER positive patients who were treated with tamoxifen for 5 years and have complete 10 years follow-up reveals that those having high expression of E2F7 have a significantly higher risk of relapse and **B.** shorter distant-relapse free survival (DFFS) time. **C.** Univariate and multivariate Cox regression model of the 134 cases including the E2F7 expression (high vs low) and all the prognostic parameters provided by the dataset indicates that E2F7 is an independent prognostic factor of DFFS. **D.** Meta-analysis of the prognostic impact of E2F7 expression. Meta-analysis results and *p*-value were presented in the forest plot. Survival time includes relapse free survival (marked with &), distant-relapse free survival (marked with @) and distant-metastasis free survival (marked with $).

To determine the role of E2F7 significance in clinical specimen, we analyzed a publicly available mRNA microarray dataset (GSE22219) [24, 25]. In this dataset, ERα positive breast cancer patients (*n* = 134) were treated with tamoxifen for 5 years and followed up for 10 years. E2F7 expression was significantly higher in the patients that had relapsed within 10-years follow-up (*n* = 49) than in patients that had not relapsed (*n* = 85) (*p* = 0.0001, Figure 5A). We also conducted a Kaplan-Meier survival analysis with samples dichotomized into 2 groups with E2F7 expression levels less than or equal to median (*n* = 67) and levels more than median (*n* = 67). The result indicated that higher expression of E2F7 is significantly associated with poor distant relapse free survival (*p* = 0.003, Figure 5B).

Next, we evaluated the correlation of E2F7 expression with clinic pathological status of the 134 patients analyzed in Figure 5A (Table 2). No significant correlation was observed between E2F7 mRNA level and age, tumor size, lymph node metastasis. However, E2F7 expression was significantly associated with tumor grade (*p* = 0.041) and strongly associated with distant relapse free survival (*p* = 0.007). More importantly, E2F7 could serve as an independent prognostic factor of distant relapse free survival time for those 134 patients in a multivariate Cox regression analysis of all the available clinic pathological factors of the GSE22219 dataset (*p* = 0.0033, HR=2.51 95% CI: 1.36-4.64, Figure 5C).

**Table 2:** Correlation of E2F7 mRNA expression with clinico-pathological indicators in GSE22219 dataset

Parameter	E2F7 Low	E2F7 High	χ2
Age (years)			*p* = 0.0814
<60	43 (57.33%)	32 (42.67%)	
> = 60	24 (40.68%)	35 (59.32%)	
Tumour size (cm)			
<2	24 (57.14%)	18 (42.86%)	*p* = 0.3519
> = 2	43 (46.74%)	49 (53.26%)	
Lymph node			
Negative	40 (51.28%)	38 (48.72%)	*p* = 0.8611
Positive	27 (48.21%)	29 (51.79%)	
Tumour grade			*p* = 0.041
1	20 (66.67%)	10 (33.33%)	
2	31 (48.44%)	33 (51.56%)	
3	9 (33.33%)	18 (66.67%)	
Distant-relapse event			*p* = 0.0007
No-relapse	52 (61.18%)	33 (38.82%)	
Relapse	15 (30.61%)	34 (69.39%)	

To further validate the prognostic value of E2F7 expression in a large population of breast cancer patients receiving tamoxifen, we did a meta-analysis of all the publicly available datasets that have complete survival data and clearly indicated the use of tamoxifen. Five datasets (GSE6532-plus2, GSE6532-U133B [26], GSE9195 [27], GSE3494 [28], GSE58644 [29]), together with GSE22219, were chosen after screening GEO and ArrayExpress websites. Some other datasets were not chosen because their microarray had no E2F7 probe or the qualified patients were less than 30. Kaplan-Meier survival analysis of each dataset indicated that E2F7 high expression is significantly associated with poor relapse free survival after tamoxifen treatment in 3 of 6 datasets (*p* < 0.05, Figure 5B, Figure S6). The result of fixed effect model (*p* < 0.0001, HR = 1.83, 95%CI: 1.38-2.45) and random effect model (*p* = 0.0008, HR = 1.87, 95% CI: 1.30-2.71) of meta-analysis all indicated a significant prognostic impact of the E2F7 expression in a total of 696 tamoxifen-treated breast cancer patients (Figure 5D).

In conclusion, high E2F7 expression is significantly correlated with relapse risk and relapse free survival in a large population and may serve as an independent prognostic factor in breast cancer patients receiving tamoxifen.

## DISCUSSION

In this study, we developed two *in vitro* tamoxifen resistant cell lines and performed a miRNA expression profile - functional screening on MCF7 cell line. We identified two down-regulated and functionally relevant miRNAs, miR-15a and miR-497. Interestingly, miR-15a and miR-497 belong to the same family. Our further investigation indicated that most of miR-15a family members, especially miR-15a/16, were reduced in the MCF7-Re cells and exogenous overexpression of miR-15a/16 re-sensitized MCF7-Re and T47D-Re cells to tamoxifen by inducing cell cycle arrest and apoptosis. Those function were largely dependent on Bcl-2 and Cyclin E1, which were associated with tamoxifen resistance in previous studies [30, 31].

MiR-15a/16 are known to act as tumor suppressors by targeting multiple oncogenes. MiR-15a/16 were frequently deleted in CLL [32] and their downregulation has been reported in a variety of cancers, such as pituitary adenomas, and prostate carcinoma [33]. Exogenous expression of these miRNAs inhibited cell proliferation, promoted apoptosis, and suppressed tumorigenicity both *in vitro* and *in vivo* [9]. However, in most cancers, mechanisms underlying the downregulation of miR-15a/16 cluster remained illusive. Investigation of the upstream mechanisms how miR-15a/16 cluster is silenced is important for developing appropriate targets or drugs to overcome problems such as tamoxifen resistance. We now found that E2F7 was responsible for the repression of miR-15a/16 cluster by competing with E2F1 for binding to the promoter of miR-15a/16 host gene DLEU2 in tamoxifen resistant breast cancer cells. It was also reported that E2F6 inhibited the expression of Apaf-1 via competing with E2F1 for the E2F-consensus site of Apaf-1 promoter in K562 cells [34]. Our study provides another evidence for the mechanism that repressive E2Fs could inhibit the transcription of E2F target genes by directly competing with active E2Fs.

Development of more specific biomarkers that predict therapeutic response to tamoxifen therapy is one of the major challenges for the successful treatment of breast cancer [1]. Re-analysis of microarray data is a powerful method to study the association between gene expression levels and clinic-pathological parameters in published datasets [35]. After re-analyzing E2F7 mRNA expression data in all 134 ER positive patients of GSE22219 dataset, we found that highly expressed E2F7 is significantly associated with high risk of relapse and E2F7 expression level can serve as an independent prognostic indicator. Additionally, meta-analysis of 696 tamoxifen-treated patients from multiple datasets strongly suggests that high expression of E2F7 is significantly associated with poor prognosis after tamoxifen therapy. This data corroborated that E2F7 may be an independent prognosis indicator in ER positive breast cancer patients receiving tamoxifen.

In summary, we found that miR-15a/16 are critical in tamoxifen resistance of breast cancer. Further analysis of its targets and upstream regulatory mechanism showed that highly expressed E2F7 represses miR-15a/16 expression, which then up-regulates Bcl-2 and Cyclin E1 to induce tamoxifen resistance of breast cancer cells. Analysis of multiple independent datasets indicated that E2F7 is significantly associated with the prognosis of breast cancer patients receiving tamoxifen. High expression of E2F7 may be developed as an independent marker for poor prognosis in breast cancer patients receiving tamoxifen and targeting E2F7 could be an effective strategy to overcome tamoxifen resistance.

## MATERIALS AND METHODS

### Cell culture and reagents

The human breast cancer MCF7 and T47D cell line was obtained from the American Type Culture Collection (Manassas, VA, USA). MCF7-Pa cells were cultured in DMEM supplemented with 10% fetal bovine serum (FBS) and 40U/ml insulin. T47D-Pa cells were cultured in RPMI-1640 supplemented with 10% FBS. Tamoxifen resistant sublines (MCF7-Re and T47D-Re) were maintained in 1640 medium without phenol red (Life Technologies, USA) supplemented with 5% charcoal-stripped FBS (cFBS) (HyClone, USA) and 1 uM 4-hydroxytamoxifen (Sigma-Aldrich, MO, USA) according to previous reported studies [15, 16]. Before transfection and analysis, parent cells and tamoxifen resistant cells were cultured in RPMI-1640 medium without phenol red supplemented with 5% cFBS and without tamoxifen at least 4 days. All of the cell lines were maintained in a humidified atmosphere containing 5% CO_2_ at 37°C.

4-hydroxytamoxifen was purchased from Sigma-Aldrich (MO, USA) and dissolved in ethanol. MiRNA mimics, miRNA ASO and siRNAs were purchased from RiboBio (Guangzhou, China). The siRNA sequences targeting to E2F7 and E2F1 in this study were chosen from previous report [36].

### MiRNA microarray analysis

Affymetrix^®^ GeneChip^®^ miRNA Array 3.0 was conducted to detect the expression of miRNAs in MCF7-Pa and MCF7-Re cells. After cultured in RPMI 1640 medium without phenol red supplemented with 5% cFBS and without tamoxifen, total RNA was harvested using TRIzol^®^ reagent (Life Technologies, USA) according to the manufacturer's instructions. RNA extraction and miRNA profile detection was provided by CapitalBio Corporation (Beijing, China). Raw data were analyzed using expression console software version 1.2 using default analysis settings and RMA as normalization method. MiRNAs with values above the cutoff of 10 in all samples were chosen for data analysis. Differentially expressed miRNAs were identified through Fold Change filtering. Microarray data were deposited to GEO database with the accession code GSE66607.

### Cell viability, apoptosis and cell cycle assay

Cells transfected with miRNA mimics or ASOs or siRNAs and treated with 1 uM tamoxifen for 3 or 4 days. Cell viability was measured by 3-(4, 5-Dimethylthiazol-2-yl)-2,5-diphenyltetrazolium bromide (MTT) assay (Sigma-Aldrich, MO, USA). Cells for apoptosis were harvested and stained with Annexin V-PI apoptosis kit (Multisciences Biotech, Hangzhou, China) according to manufacturer's instructions. For cell cycle analysis, cells were harvested and fixed in 70% ethanol for one night and stained with propidium iodide (PI). Apoptosis and cell cycle were detected using Accuri™ C6 flow cytometer (BD Biosciences, CA, USA). The fractions of cells in each phase of cell cycle (G0/G1, S and G2/M, as indicated as % cells) were determined using Flowjo 7.6 cell cycle analysis software (Tree Star, San Carlos, CA).

### Library screening for miRNA

MCF7-Re cells were cultured in RPMI 1640 medium without phenol red supplemented with 5% cFBS and without tamoxifen for 7days and seeded in 96-well plates (5000 cells per well). MiRNA mimics library was purchased from CapitalBio Corporation (Beijing, China). MCF7-Re cells were transfected with miRNA mimics using Lipofectamine^®^ 3000 (Life Technologies, USA). Transfections were carried out in 96-well plates with opti-MEM medium (Life Technologies, USA). After 8 hours transfections, opti-MEM medium was replaced wiht RPMI 1640 medium without phenol red supplemented with 5% cFBS and 1 uM tamoxifen. Cells viability were measured by MTT after 48 hours of transfection.

### Construction of plasmids and Luciferase activity assay

Potential upstream promoter regions of DLEU2 were amplified using PCR and cloned into the pGL3-basic vector (Promega, Madison, WI) as described previously [37]. A series of deleted promoter fragment and E2F binding site mutant promoter fragment were amplified using PCR and cloned into the pGL3-basic vector [37]. Dual luciferase reporter assay was performed using Dual-luciferase Reporter Assay System (Promega, Madison, WI) according to manufacturer's instructions.

### Quantitative RT-PCR (qPCR)

The total RNA was extracted using TRIzol (Life Technologies, USA) according to the manufacturer's instructions. The reverse transcription was performed using transcriptase (Life Technologies, USA), and the real-time PCR was performed in a LightCycler480 System using a SYBR Premix ExTaq kit (Takara, Shiga, Japan). Bulge-Loop™ miRNA qPCR Primer Sets purchased from RioboBio (Guangzhou, China) were used in the mature miRNA reverse transcription and qPCR. U6 small nuclear RNA was used as an internal control.

### Chromatin immunoprecipitation (CHIP) assay

CHIP and following qPCR were performed using Agarose ChIP Kit (Thermo Fisher Scientific, California, USA) or ChIP-IT^®^ Express Enzymatic CHIP Kit (Active Motif, Carlsbad, USA) according to manufacturers' instructions. Chip-grade primary antibodies against E2F7 (Santa Cruz biotechnology, USA) and E2F1 (Cell Signaling Technology, MA, USA) were used in the chip experiments and a normal rabbit IgG (Santa Cruz biotechnology, USA) was served as a negative control. DLEU2 promoter chip promoter: F. GCGGGGTTGGCTCTAACGAAT; R. GGTTATCCTGTCTCTCCCGCT

### Western blot analysis

Western blot analysis was performed as described previously [37]. The β-actin antibody (ProteinTech, Wuhan, China), Bcl-2 (Santa Cruz biotechnology, USA), Cyclin E1 (Santa Cruz biotechnology, USA), E2F1 (Cell Signaling Technology, MA, USA), E2F7 (Santa Cruz biotechnology, USA) were all used according to the manufacturers' instructions. HRP-cojugated secondary antibodies were purchased from ProteinTech (Wuhan, China). All blots were detected using the enhanced chemiluminescence (ECL) with ChemiDoc™ XRS+ imaging system (Bio-Rad, CA, USA). Images were analyzed with Image Lab™ Software (Bio-Rad, CA, USA).

### Statistical analysis

Data were presented in terms of means and standard errors for at least three separate experiments. Two tail Student's *t* test was used to compare two treatment groups. Graph Pad Prism version 5 (GraphPad software Inc., CA, USA) was used for statistical analysis. The results were considered statistically significant when *p* is less than 0.05.

### Analysis of patient data

A dataset with 134 ER positive patients who were treated with tamoxifen for 5 years and have complete 10 years follow-up information was obtained from the NCBI GEO database (GEO Accession GSE22219). The expression of E2F7 mRNA expression values were obtained from processed data and were compared between patients who had relapsed after surgery using two tail Student's *t* test. 134 ER positive patients were equally divided into two groups based on the E2F7 mRNA level and Kaplan-Meier survival curve was carried out in Graph Pad prism software and between groups comparison was performed using the log-rank test. A chi-square test was used to analyze the association between E2F7 mRNA expression levels and clinicopathological characteristics, the result was shown in table 2. The survival data were then evaluated using univariate and multivariate Cox regression model on MedCalc software for windows, version 14.12.0 (MedCalc Software, Ostend, Belgium) as described previously [38]. Significant prognostic parameters (*p* < 0.05) were used to evaluate in multivariate Cox regression model.

The meta-analyses were carried out using the publicly available statistical computing language R (version R-3.1.1) as described previously [17]. Briefly, 696 patients of 6 independent datasets were involved after a comprehensive search in available databases. Hazard ratio (HR) of every dataset was calculated on MedCalc software independently. E2F7 expression level was defined by the probe set intensity and if there were two probe sets match the E2F7 mRNA in one microarray platform, we took the average value of the two probe sets. Fixed effect models and random effects models of the R package “meta” were used based on parameter estimates of log HRs in Cox models and their SEs [39]. Results were visualized with forest plots, in which parameter estimates of all single studies and the pooled estimates along with their confidence intervals are plotted on top of each other. Adjustment for multiple testing was conducted with the method of Benjamini and Hochberg [40].

## SUPPLEMENTARY FIGURES


