# Supplementary material for: E2F7 overexpression leads to tamoxifen resistance in breast cancer cells by competing with E2F1 at miR-15a/16 promoter
Source: Oncotarget. 2015 Sep 12;6(31):31944–57. doi: 10.18632/oncotarget.5128 (PMC4741652; doi:10.18632/oncotarget.5128)
Supplement: Supplementary file 1 [file oncotarget-06-31944-s001.pdf]

SUPPLEMENTARY FIGURES

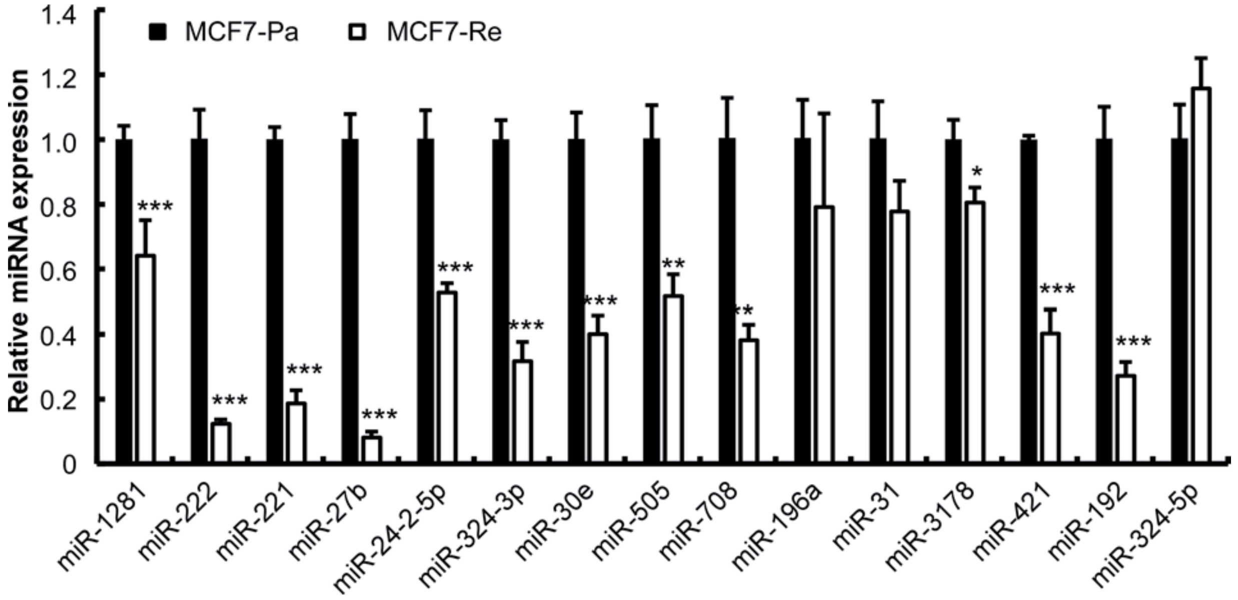

Supplementary Figure S1: Verification of down-regulated miRNAs identified by microarray in MCF7-pa and MCF7-Re cells. Expression of other 15 mature miRNAs were determined in MCF7-Pa and MCF7-Re cells by qPCR. (*p < 0.05, **p < 0.01, ***p < 0.001.)

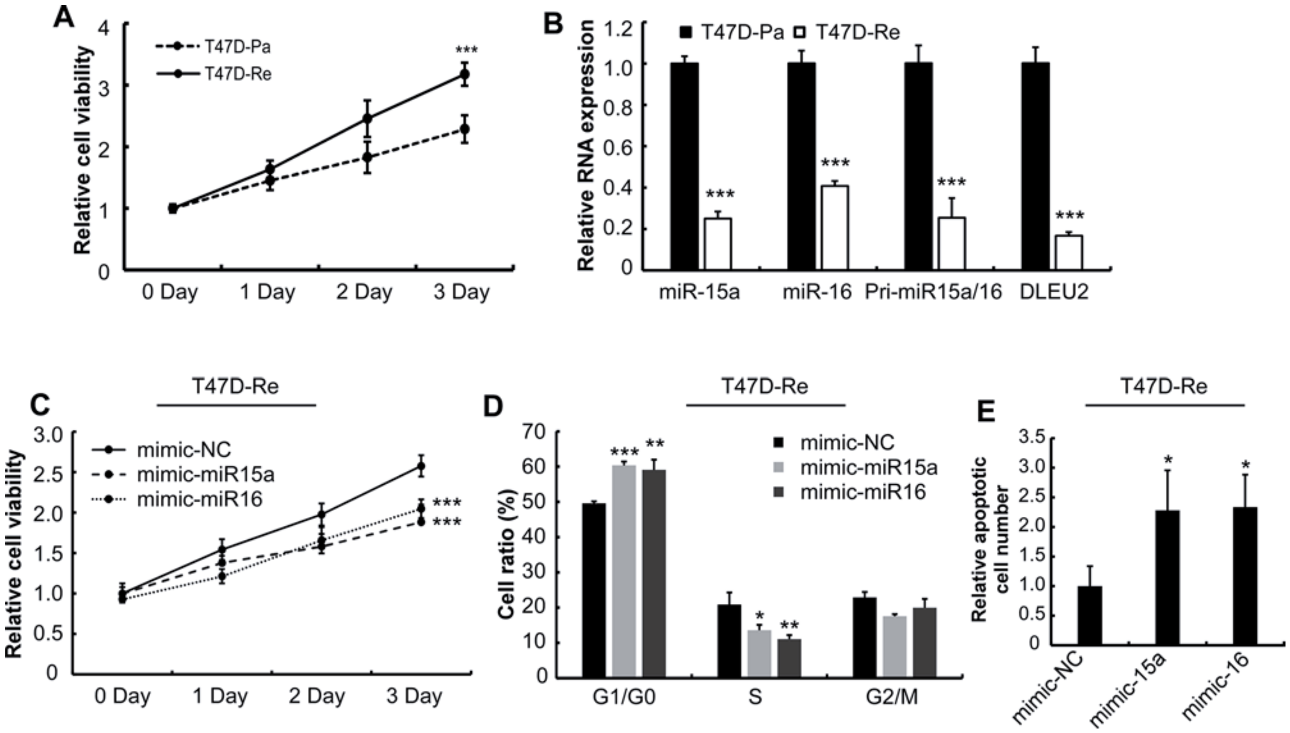

Supplementary Figure S2: MiR-15a/16 regulate tamoxifen resistance in T47D cell line. A. Proliferation of T47D-Pa and T47D-Re were determined by MTT under 1 uM Tamoxifen treatment. **B.** Expression of mature miR-15a/16, pri-miR15a/16 and host gene DLEU2 in T47D-Pa and T47D-Re cells were detected by qPCR. U6 was used as an internal control for mature miRNA and β-actin mRNA was used as an internal control for primary miRNA and DLEU2 RNA. **C.** T47D-Re cells proliferation were determined by MTT after transfected with miRNA mimics under 1 uM tamoxifen. Cell cycle **D.** and apoptosis **E.** were measured after 3 days transfection and treatment with 1 uM tamoxifen. (*$p < 0.05$, **$p < 0.01$, ***$p < 0.001$.)

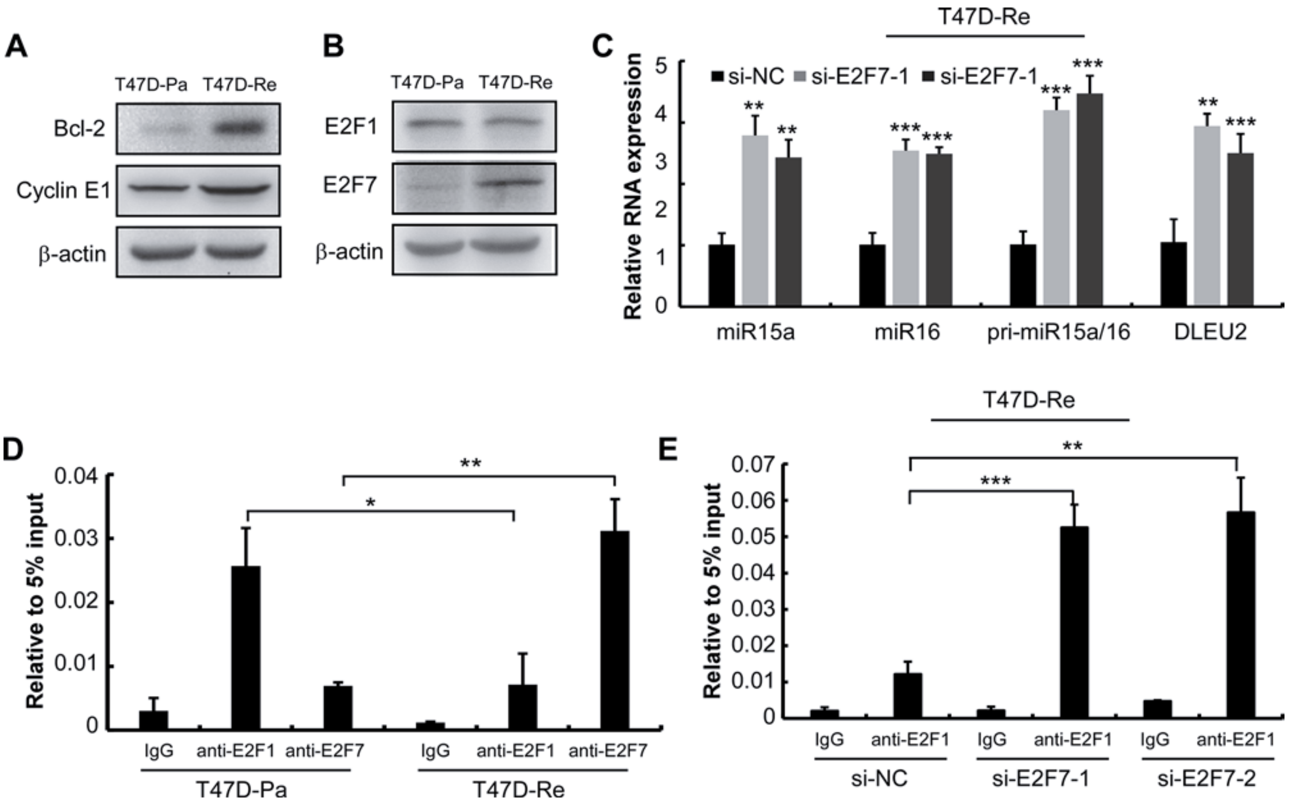

Supplementary Figure S3: E2F7 regulates miR-15a/16 expression in T47D-Re cells. A. Western blot analysis for Bcl-2 and Cyclin E1 in T47D-Pa and T47D-Re cells. β-actin was used as an internal control, hereafter. **B.** Western blot analysis for E2F1 and E2F7 in T47D-Pa and T47D-Re cells. **C.** qPCR of mature miR15a, miR16, pri-miR15a/16 and DLEU2 in T47D-Re cells after transfected with E2F7 siRNAs for 48hours. **D.** ChIP was performed to measure the binding activity of E2F1 and E2F7 to DLEU2 promoter in T47D-Pa and T47D-Re cells. Primer was designed to detect predicted E2F binding site. Data are normalized to 5% input for each cell type. **E.** CHIP was performed to measure the binding activity of E2F1 to DLEU2 promoter after 48hours of transfection of E2F7 siRNAs in T47D-Re cells. (**p* < 0.05, ***p* < 0.01, ****p* < 0.001.)

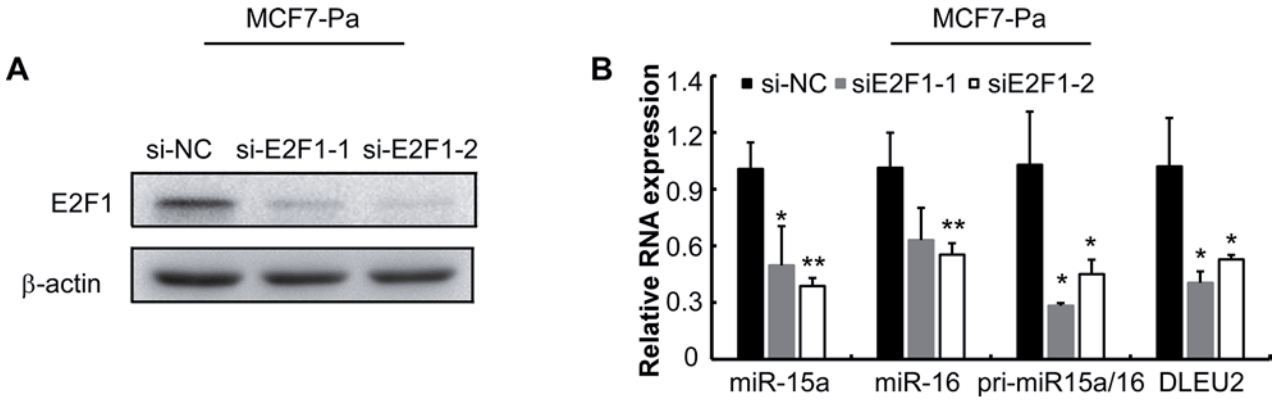

Supplementary Figure S4: Knocking down E2F1 represses miR15a/16 expression in MCF7-Pa cells. **A.** Expression of E2F1 protein was detected by western blotting after 48 hours of transfection of E2F1 siRNAs in MCF7-Pa cells. **B.** Expression of mature miRNA-15a, miRNA-16, primary miRNA-15a/16-1 and their host gene DLEU2 were detected by qPCR after 48 hours of transfection of E2F1 siRNAs in MCF7-Pa cells. U6 was used as an internal control for mature miRNA and β-actin mRNA was used as an internal control for primary miRNA and DLEU2 RNA. (*$p < 0.05$, **$p < 0.01$)

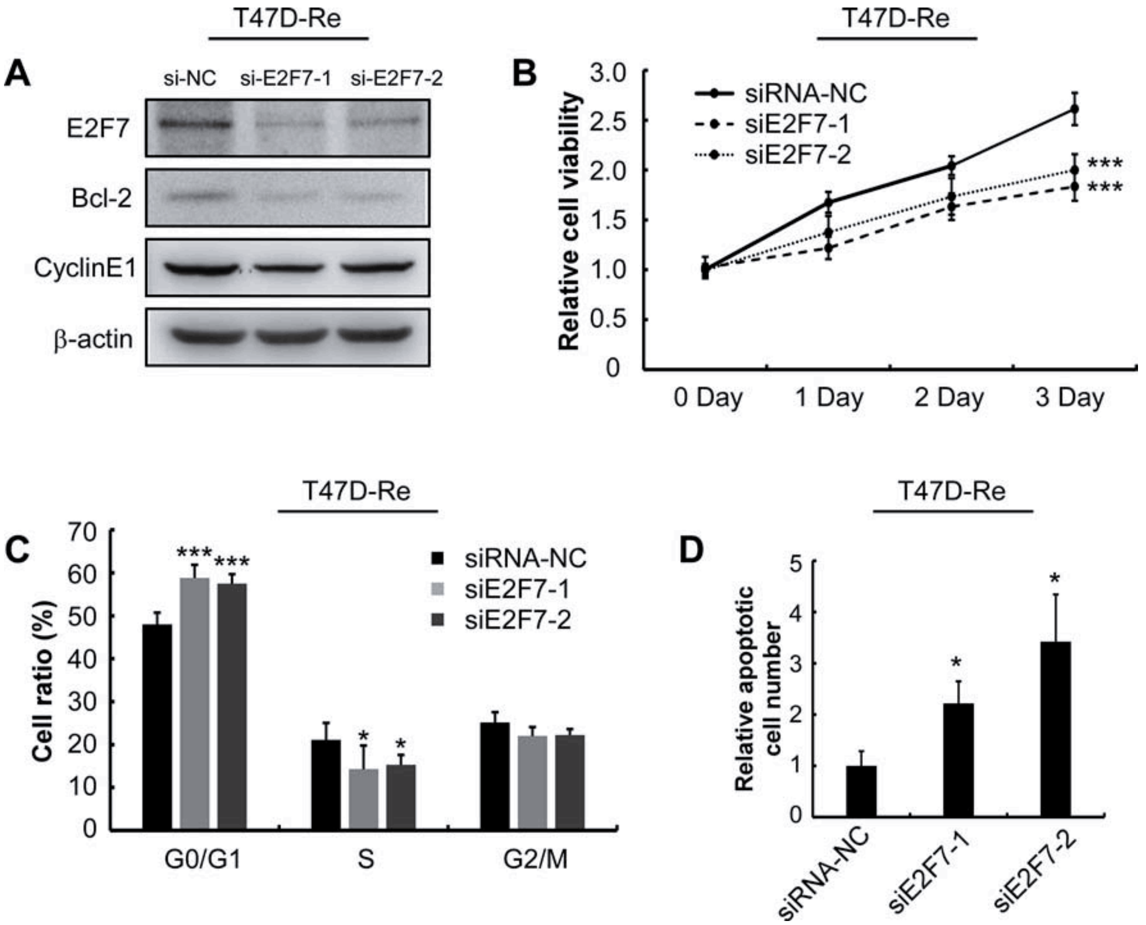

Supplementary Figure S5: Silencing of E2F7 in T47D-Re cells sensitizes cells to tamoxifen. A. E2F7, Bcl-2 and Cyclin E1 protein expression was determined by western blotting in T47D-Re cells that were transfected with E2F7 siRNAs. **B.** proliferation rate of T47D-Re cells under 1 uM tamoxifen were measured every day after transfected with E2F7 siRNAs by MTT assay. The absorbance value of 0 day was taken as 1. (***$p < 0.001$ versus negative control siRNA of the 3rd day). **C.** T47D-Re cells were transfected with E2F7 siRNAs and treated with 1 uM tamoxifen for 3 days and cell cycle distribution was monitored by flow cytometry, **D.** apoptosis was detected by flow cytometry after Annex-V and PI staining. (*$p < 0.05$, ***$p < 0.001$)

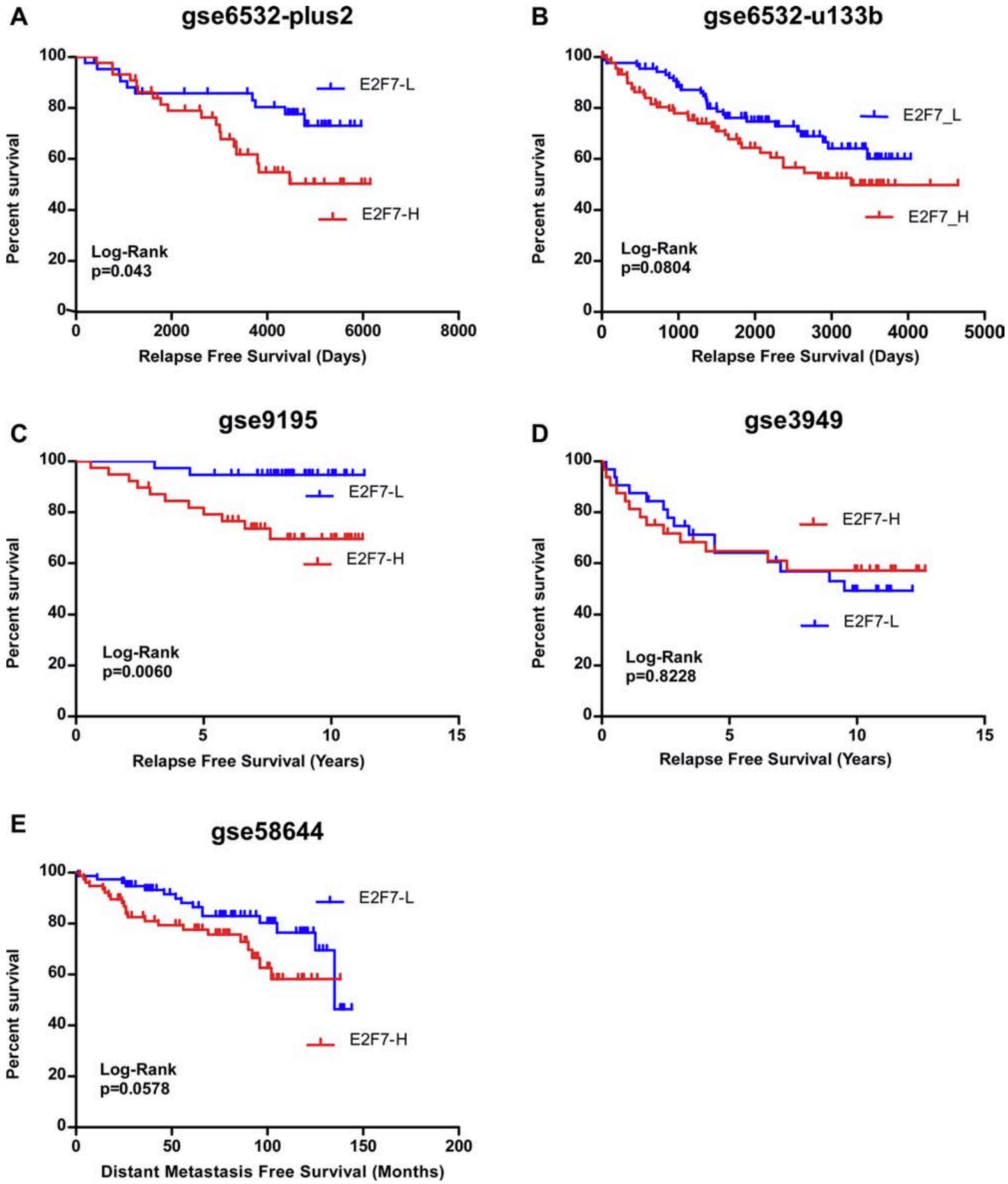

Supplementary Figure S6: Kaplan–Meier survival analysis of the other 5 datasets. **A–E.** Kaplan–Meier progress free survival analysis of E2F7 mRNA expression in tamoxifen treated breast cancer patients from 5 datasets. Log Rank *p* value of each dataset was shown in survival curve.
